# Far from easy and accurate - detection of metabolic syndrome by general practitioners

**DOI:** 10.1186/1471-2296-10-76

**Published:** 2009-11-30

**Authors:** Eeva-Eerika Helminen, Pekka Mäntyselkä, Irma Nykänen, Esko Kumpusalo

**Affiliations:** 1 Kuopio Health Centre, Kuopio, Finland; 2 Family Practice Unit, Kuopio University Hospital, Kuopio, Finland; 3 School of Public Health and Clinical Nutrition, University of Kuopio, Kuopio, Finland

## Abstract

**Background:**

Metabolic syndrome (MetS) is a major public health challenge. General practitioners (GPs) could play a key role in its recognition. However, it often remains undiagnosed in primary care. This study assesses how well GPs and patients recognise MetS among patients with coronary heart disease or at least one of its risk factors.

**Methods:**

Twenty-six health centres around Finland were randomly selected for the purpose of identifying, over a two-week period in April 2005, patients meeting the inclusion criteria of coronary heart disease or one of its risk factors. GPs and identified patients (n = 1880) were asked to complete surveys that included a question about the patient's MetS status. A trained nurse conducted health checks (n = 1180) of the identified patients, utilising criteria of MetS modified from the National Cholesterol Program. Data from the GPs' survey were compared with those from the health check to establish the extent of congruence of identification of MetS.

**Results:**

Almost half (49.4%) of the patients met the criteria of MetS as established by objective measures. However, in the GPs' survey responses, only 28.5% of the patients were identified as having MetS. Additionally, these groups of MetS patients were not congruent. The sensitivity of the GPs' diagnosis of MetS was 0.31 with a specificity of 0.73. Only 7.1% of the study patients stated that they were suffering from MetS.

**Conclusion:**

Detection of MetS is inaccurate among GPs in Finland. Most patients were not aware of having MetS. The practical relevance of MetS in primary care should be reconsidered.

## Background

Because of the epidemic of overweight and sedentary lifestyle, the prevalence of metabolic syndrome (MetS) is increasing worldwide [1], and the syndrome has become a major public health challenge [2]. General practitioners (GPs) play a key role in recognising MetS, but it is rarely recorded as a diagnosis in clinical practice [3]. Despite several efforts to make diagnosis feasible for clinicians, the syndrome often remains undiagnosed in primary health care [4].

During the last decade, various medical organizations have published their criteria of metabolic syndrome [5]. However, only the two most recent definitions are suitable for the primary care framework. In 2001 the National Cholesterol Education Program (NCEP) published a working definition of the syndrome based on five commonly measured clinical criteria that physicians could implement in their practices [6]. In 2005 the International Diabetes Federation (IDF) released their set of criteria of MetS [7]. The IDF definition provides a stepwise approach to risk, with measurement of the waist as a simple initial screening test followed by assessment of four other components.

Finland has been a pioneer in promoting the concept of MetS since the beginning of the nineties [8]. The leading domestic professional journal for doctors in Finland first published articles about this topic in 1992 [9]. MetS has been represented in the Finnish Physician's Handbook since 1996 [10]. The Handbook has portrayed MetS as a risk factor of cardiovascular diseases and has described the epidemiology and clinical features of MetS. The Finnish version of ICD-10 has had a code for MetS (E66.00) since 1999. Moreover, since the early 2000s, there has been a steady stream of information about MetS for GPs, provided mainly by specialists and pharmaceutical companies. Based on these efforts and scientific literature, MetS can be considered to constitute a very common and important risk factor of diabetes and cardiovascular diseases. This means primary care physicians should have the most important role in detecting and treating MetS. However, it remains unclear to what extent MetS is recognised by GPs.

In this study we examined how well GPs recognise MetS among patients with coronary heart disease (CHD) or at least one of its risk factors (previously diagnosed diabetes or metabolic syndrome, hypertension, smoking or dyslipidaemia). We compared these results with the actual percentage of patients meeting the criteria of MetS according to modified NCEP criteria (Table 1). We also established the extent to which the patients themselves were aware that they had MetS.

**Table 1:** Heart 2005 study criteria of metabolic syndrome according to modified National Cholesterol Education Program criteria.

Metabolic syndrome is present when three or more of the five criteria are met.	
1. impaired glucose tolerance (IGT), increased fasting plasma glucose (IFG) or diabetes (DM)	
2. hypertension ≥140/90 mmHg or medication for hypertension	
3. serum triglyceride concentration ≥1.7 mmol/l	
4. reduced serum HDL cholesterol < 1.2 mmol/l for women and < 1.0 mmol/l for men	
5. waist circumference > 88 cm for women and > 102 cm for men.	

HDL, high density lipoprotein.

## Methods

### Participants

The Heart 2005 study was carried out in 26 randomly selected primary care health centres around Finland that represented the entire Finnish public primary care system in terms of size and location. Altogether 181 general practitioners collected the data during two workweeks in April 2005. The study was a descriptive cross-sectional study, and it was approved by the Ethics Committee of Kuopio University Hospital and the University of Kuopio.

The patients included in the study had to have CHD or at least one of its risk factors, such as previously diagnosed diabetes or metabolic syndrome, hypertension, smoking or dyslipidaemia. A patient was considered to have CHD if it had been diagnosed by a doctor or it was indicated by a specific code on the patient's health insurance card. The study doctors made records of CHD and the risk factor status of each patient. All the patients who visited the health centre during the two weeks and met the study criteria were registered and invited to attend a health check conducted by a trained nurse.

From the regular patient stream, 1880 patients were identified as meeting the inclusion criteria of the study. Written informed consent to participate in the study was given by 1331 of them. Altogether 1208 filled in the patient questionnaire and 1180 of them attended the health check. Thus, the participation rate was 62.8%. The basic characteristics and measurements of the study patients are shown in Table 2.

**Table 2:** Basic characteristics of 1160 Heart 2005 study subjects.

Age (years) n = 1159	63.8 (11)
≥ 65 years (%) n = 1159	53.9
Sex (men %) n = 1135	42.8
BMI n = 1155	29.7 (5.1)
Current smokers (%) n = 1152	16.4
Hypertension (%) n = 1116	73.8
Dyslipidaemia (%) n = 1109	69.7
CHD (%) n = 1080	19.4
Diabetes (%) n = 1083	28.4
Fasting plasma glucose (mmol/l) n = 1042	6.15 (1.5)
SBP (mmHg) n = 1136	144 (19)
DBP (mmHg) n = 1135	84 (10)
Total cholesterol (mmol/l) n = 1085	5.11 (1.0)
Triglycerides (mmol/l) n = 1050	1.47 (0.80)
HDL cholesterol, men (mmol/l) n = 437	1.3 (0.38)
HDL cholesterol, women (mmol/l) n = 599	1.6 (0.47)
Waist circumference, men (cm) n = 465	105 (12)
Waist circumference, women (cm) n = 631	95 (14)

BMI, body mass index; CHD, coronary heart disease; SBP, systolic blood pressure; DBP, diastolic blood pressure; HDL, high density lipoprotein.

Figures are means (SD) unless stated otherwise.

The GPs collecting the patient data were mainly senior doctors, and 67.5% of them had over 10 years of work experience. The average age of female doctors was 42 years and of male doctors, 47 years. There was a slight predominance of female doctors (56.1%).

### Data collection

At the health check, after a five-minute rest the nurse measured the patient's blood pressure twice with an interval of a few minutes, using a cuff fitting the patient's upper arm circumference. The mean of the measurements was used in analysing the results. The nurse recorded the latest laboratory results of haemoglobin, total serum cholesterol, HDL and LDL cholesterol and triglycerides as well as fasting plasma glucose and Hb-A1c values. If these tests had not been conducted during the last 12 months, the patient was sent to the laboratory. The nurse also measured the patient's height, weight and waist circumference.

The patients agreeing to take part in the study were asked to fill in a questionnaire. One question asked whether the patient had ever been diagnosed with or treated for metabolic syndrome, with the possible answer being "yes" or "no". The GPs filled in a doctor's questionnaire about every patient registered for the study, and again one question asked whether the patient had metabolic syndrome, the answer being either "yes" or "no".

The criteria of metabolic syndrome used in the study were given in the doctor's questionnaire (Table 1). The criteria followed the NCEP definition [6], with some modifications. Impaired glucose tolerance and diabetes were added to the first criterion. There was a higher threshold value for hypertension and the use of antihypertensive medication was included in the second criterion. Also, the cut-off values were slightly lower for HDL cholesterol. These modifications were made according to the criteria of MetS in the Finnish Physician's Handbook 2004 [11]. The threshold value used for increased fasting plasma glucose was ≥5.6 mmol/l.

The data collected in the patient's questionnaire, in the doctor's questionnaire and at the health check were used to analyse how many of the patients met the study criteria of MetS. The laboratory results collected at the health check were used to determine whether the patient met each criterion according to the threshold values indicated in Table 1. The patient was considered to have diabetes if the question "Have you been diagnosed with diabetes?" was answered positively or if the doctor had replied positively to the question whether or not the patient had diabetes. The patients were also asked "Have you undergone an oral glucose tolerance test (OGTT)?" The possible answers were (1) "no", (2) "yes, my blood glucose levels were normal", and (3) "yes, my blood glucose levels were above normal". The patient was considered to have IGT if the third option was chosen.

### Statistical analysis

The results are expressed as frequencies. The sensitivity and specificity of the GPs' diagnosis of metabolic syndrome were calculated. All statistical analyses were performed using SPSS statistical software for Windows, version 16.0.

## Results

The GPs had answered the question about metabolic syndrome in 1173 (99.4%) of the 1180 patient cases. They assessed that 28.5% of the study patients (30.7% of the men, 27.4% of the women) had MetS according to the study criteria. However, 49.4% of the patients (50.8% of the men, 49.6% of the women) met the study criteria of metabolic syndrome according to the measurements and records made at the nurse's appointment and the answers from the patients' questionnaires. The measurements and records were collected appropriately from 1160 (98.3%) of the 1180 study patients, as 20 (1.7%) individuals had to be excluded due to missing data. Moreover, the group of patients with MetS according to the GPs' evaluation did not quite match with the group of patients actually meeting the study criteria of the syndrome. The sensitivity of the general practitioners' diagnosis for MetS was 0.31 and the specificity was 0.73 (Table 3).

**Table 3:** Metabolic syndrome reported by general practitioners versus patients meeting the study criteria of metabolic syndrome in the Heart 2005 study (n = 1153).

		MetS reported by GPs (n = 1173) (%)		
			
		Yes	No	
Patients meeting the study criteria of MetS (n = 1160) (%)	yes	176 (30.9)	394 (69.1)	570
	no	157 (26.9)	426 (73.1)	583
		333	820	1153

MetS, metabolic syndrome; GPs, general practitioners

Of the 1180 patients, 1059 (89.7%) had answered the question about whether they had been diagnosed with or treated for metabolic syndrome. In 75 (7.1%) cases the answer was positive. Of the patients reporting having MetS, 53 (70.7%) met the study criteria, as opposed to 466 subjects (48.2%) in the group reporting that they did not have the syndrome.

## Discussion

In our study, MetS was poorly detected by general practitioners; the sensitivity of diagnosis was only 0.31. Much work has been done during the last 15 years to familiarise GPs with the syndrome, but it seems that it is still unrecognised. On the other hand, the fact that only 75 (7.1%) out of 1059 patients reported having MetS suggests that the condition is even more unclear to patients.

It can be considered quite alarming that the GPs did not recognise MetS in patients meeting the criteria of the syndrome. However, there may be valid reasons for this. For example, over the years there have been several different definitions of MetS, which may well have confused rather than helped GPs in diagnosing the syndrome. We did not use as strict a definition of MetS as actual NCEP criteria. Further, some of the patients might have had IGT, resulting in more cases of MetS. Hence, the real proportion of patients with MetS could have been slightly higher. However, the definition of MetS used in the present study is comparable with the definition that was used in Finnish primary care during the study period.

Our study was conducted during the hectic everyday routine in the primary care setting. This may have affected the accuracy of MetS detection by the GPs. Nonetheless, these are the real working conditions in a GP's office. MetS has been promoted as a simple public health care strategy for doctors to identify patients at high risk [12]. If it does not work in real-life conditions, such as in the bustle of a GP's office, then it will not meet its original aim.

There might be several reasons why GPs are not using the diagnosis of MetS. Firstly, over the years more and more counter-arguments have been raised about the diagnostic, prognostic and therapeutic value of MetS [13-15]. Secondly, differing and even contradictory [15] statements from various expert organizations have been announced. On top of that, there are already several tools for identifying apparently healthy individuals at an elevated risk of diabetes, such as the glucose tolerance test [16,17] and the diabetes risk score [18], or of CVD, such as the Framingham risk score [19] or the SCORE model [20]. As the time reserved for a GP's appointment is limited, it is not realistic to assume that several risk assessment models would be used in evaluating one patient.

How is MetS going to survive in this battle of prevention strategies, and more importantly, what is the future role of MetS? Metabolic syndrome is not likely to replace currently used global risk scoring algorithms, so both traditional risk factors and emerging metabolic markers associated with metabolic syndrome should be incorporated in future risk assessment systems [21]. The need for such global risk evaluation tools is emphasized even more as waist circumference and waist-to-hip ratio have been shown to be strongly associated with death [22]. In a busy primary care clinical practice, this could mean a computerized decision support system that would be integrated into electronic patient records. This would enable the physician to make risk assessments based on a single algorithm without any particular scoring systems.

A cohesive and clear message is urgently needed from the scientific community to clarify how MetS and other risk evaluation models should be used in primary care.

## Conclusion

Detection of MetS is inaccurate among GPs in Finland. Most patients were not aware of having MetS. The practical relevance of MetS in primary care should be reconsidered.

## Competing interests

The authors declare that they have no competing interests.

## Authors' contributions

E-EH performed the statistical analysis, participated in the design of the study and drafted the paper. PM participated in the design of the study and helped in drafting the manuscript. IN participated in the design of the study and helped in drafting the manuscript. EK conceived the study and participated in the design of the study and helped in drafting the manuscript. All authors read and approved the final manuscript.

## Pre-publication history

The pre-publication history for this paper can be accessed here:

http://www.biomedcentral.com/1471-2296/10/76/prepub
